# Royal Australian and New Zealand College of Psychiatrists mood disorders clinical practice guidelines update

**DOI:** 10.1192/bjo.2021.469

**Published:** 2021-06-18

**Authors:** John Allan

**Affiliations:** Royal Australian and New Zealand College of Psychiatrists

## Abstract

**Aims:**

To provide guidance for the management of mood disorders, both depressive and bipolar disorders, based on scientific evidence supplemented by expert clinical consensus.

**Background:**

It is the EIT responsibility to monitor a patient's physical health and the effects of anti-psychotic medication for at least the first 12 months.

**Method:**

The update has been developed in a consistent manner to the 2015 guideline. The composition of the working group has remained largely the same as has the process to evaluate the evidence and synthesise the findings. To approach the update, the working group identified areas within the 2015 guideline where significant changes had occurred, for example the development of new therapies or where thinking and practice have changed and new ideas have emerged. Recommendations were reviewed in light of any new findings and evidence. As only some sections of the 2015 guideline have been updated/revised, the time taken to develop the update has been considerably shorter. Public consultation and peer review informed the final version.

**Result:**

This led us to review the mechanism in the team for arranging and reviewing these investigations.

**Conclusion:**

The mood disorders clinical practice guideline update addresses both depressive and bipolar disorders. It provides up-to-date recommendations and guidance within an evidence-based framework supplemented by expert clinical consensus.

